# Manifest Destiny of the English Race

**Published:** 1881-02

**Authors:** 


### MANIFEST DESTINY OF THE ENGLISH RACE.

Professor John Fiske spoke in Boston a few evenings ago on the "Manifest Destiny of the English Race." He said that as the result of the American revolution there are now two Englands prepared to work toward the political regeneration of mankind. The free government of the primitive Aryans has been maintained without interruption in England. On the continent of Europe it had been everywhere interrupted, impaired or lost, except in Scandinavia, and impregnable Aryan civilization struggled for many centuries to prove itself superior to the assaults of other barbarism. That struggle was transferred to America, after its discovery, and England contended for supremacy on this continent with France and Spain. That struggle proved which kind of civilization was endowed with the higher and sturdier political life. The race which succeeded became from that time the leading power in the world. Professor Fiske says that the process of change which is called civilization means primarily the substitution of a state of peace for a state of war, and that a general diminution of warfare is possible only by the union of small political groups into large groups. Europe can find peace only in the centering of the preponderating political strength in the hands of peaceful communities. The question of peace has been settled on this continent. The American Union was on a plan to prevent strife, and to aid civilization. The war for the Union was to preserve these conditions. Professor Fiske says the American system of federalism is one of the most important contributions the English race has made to the general work of civilization. He predicts that we shall have in a century a political aggregation surpassing in power any empire that has ever existed.

### SENSIBLE.

I.

Take the open air—
The more you take the better;
Follow Nature's laws
To the very letter.

II.

Let the doctor's go
To the Bay of Biscay;
Let alone the Gin,
Tobacco, and the Whisky.

III.

Freely exercise;
Keep your spirits cheerful;
Let no dread of sickness
Make you over-fearful.

IV.

Eat the simplest food,
Drink the pure cold water;
Then you will be well,
Or at least you ought ter.

